# Functional recapitulation of transitions in sexual systems by homeosis during the evolution of dioecy in *Thalictrum*

**DOI:** 10.3389/fpls.2013.00487

**Published:** 2013-11-27

**Authors:** Nicole C. LaRue, Alessandra M. Sullivan, Verónica S. Di Stilio

**Affiliations:** Department of Biology, University of WashingtonSeattle, WA, USA

**Keywords:** B class genes, PISTILLATA, VIGS, RNAi, ranunculid, ABC model, sex determination, MADS box genes

## Abstract

Sexual systems are highly variable in flowering plants and an important contributor to floral diversity. The ranunculid genus *Thalictrum* is especially well-suited to study evolutionary transitions in sexual systems. Homeotic transformation of sexual organs (stamens and carpels) is a plausible mechanism for the transition from hermaphroditic to unisexual flowers in this lineage because flowers of dioecious species develop unisexually from inception. The single-copy gene *PISTILLATA* (*PI*) constitutes a likely candidate for rapid switches between stamen and carpel identity. Here, we first characterized the expression pattern of all B class genes in the dioecious species *T. dioicum*. As expected, all B class orthologs are expressed in stamens from the earliest stages. Certain *AP3* lineages were also expressed late in sepal development. We then tested whether orthologs of *PI* could potentially control sexual system transitions in *Thalictrum*, by knocking-down their expression in *T. dioicum* and the hermaphroditic species *T. thalictroides*. In *T. dioicum*, we found that *ThdPI-1/2* silencing caused stamen primordia to develop into carpels, resulting in male to female flower conversions. In *T. thalictroides*, we found that *ThtPI* silencing caused stamen primordia to develop into supernumerary carpels, resulting in hermaphroditic to female flower conversions. These phenotypes illustrate the ability for homeotic mutations to bring about sudden and potentially adaptive changes by altering the function of a single gene. We propose a two-step evolutionary model where transitions from hermaphroditic to unisexual plants in *Thalictrum* result from two independent mutations at a B class gene locus. Our *PI* knockdown experiments in *T. thalictroides* recapitulate the second step in this model: the evolution of female plants as a result of a loss-of-function mutation in a B class gene.

## Introduction

*Thalictrum* is an ideal genus in which to test evolutionary transitions in sexual system: its almost 200 species display hermaphroditism, dioecy, andromonoecy (male and hermaphroditic flowers on one plant) and gynomonoecy (female and hermaphroditic flowers on one plant; Boivin, 1944; Guzmán, 2005). A molecular phylogeny of the genus has enabled ancestral character state reconstructions of sexual systems that depict two independent origins of dioecy from hermaphroditism, and a close relatedness of dioecious clades to andromonoecious or structurally androdioecious species (separate male and hermaphroditic plants, Soza et al., 2012). “Structural androdioecy” refers to plants that have the appropriate sexual organs (stamens only, or stamens and carpels), in spite of being functionally dioecious [due to sterile inaperturate pollen in hermaphrodites (Kaplan and Mulcahy, 1971; Penny and Steven, 2009)]. In the dioecious species *Thalictrum dioicum*, sex expression is stable throughout the life of a plant and male to female sex ratios are 1:1, implying a strong genetic determination of sex (Di Stilio et al., 2005). Sex ratios resulting from crosses among species with different sexual systems suggest that males are heterogametic and, in the absence of cytogenetic evidence for sex chromosomes, *Thalictrum* is described as having homomorphic sex chromosomes (Westergaard, 1958).

At a developmental level, *Thalictrum dioicum* constitutes an excellent system to test the role of organ identity genes in sex determination. The majority of angiosperms have flowers that develop unisexually through differential abortion of reproductive organs (Type I). In these flowers, sex determination is expected to act downstream of organ identity. In contrast, other flowers develop unisexually from inception (Type II), either via homeosis (e.g., *Thalictrum*, Di Stilio et al., 2005) or by lack of initiation of primordia (e.g., *Spinacia*, Sherry et al., 1993; Mitchell and Diggle, 2005). The genetic mechanisms of sex determination in species with Type II flowers are expected to act at or upstream of organ identity. Moreover, across angiosperms, the stage of reproductive organ abortion (including lack of organ abortion, defined as stage 0) is positively correlated among male and female flowers of dioecious species, pointing to a shared single regulator for both processes (Diggle et al., 2011). In conclusion, *Thalictrum dioicum* has type II flowers by homeosis whose differential development is potentially under the control of a single gene involved in stamen identity.

Despite multiple advantages offered by *Thalictrum* for evo-devo studies, it has not been possible to perform loss and gain of function experiments in the past due the absence of a stable transgenic system. Recently, however, *Thalictrum* has joined the ranks of emerging model systems in plant evo-devo where functional approaches have been enabled by virus induced gene silencing (VIGS, Dinesh-Kumar et al., 2003; Di Stilio et al., 2010; Di Stilio, 2011; Galimba et al., 2012). The next logical step is to experimentally test the homeotic sex determination model using VIGS.

Among the flower organ identity genes of the classical ABC model of flower development (Bowman et al., 1991), the B class genes *APETALA-3* (*AP3*) and *PISTILLATA* (*PI*) are logical candidates for sexual transitions. In the hermaphroditic model plant A*rabidopsis thaliana*, B class gene knockouts are effectively female, since carpeloid organs develop in place of stamens (Bowman et al., 1989), whereas B class gene overexpression lines are effectively male, with the central carpel primordia replaced by additional stamens (Krizek and Meyerowitz, 1996). In addition, mutations in either *AP3* or *PI* result in equal, full B class loss of function phenotypes (Bowman et al., 1991).

Three lineages of *AP3* were previously described in the Ranunculaceae (Kramer et al., 2003), two are present in *Thalictrum* (*ThdAP3-1* and *ThdAP3-2a/b*, Di Stilio et al., 2005). The third lineage (*AP3-3*) is expressed in petals in other members of the family (Kramer et al., 2003) but not in *Thalictrum*, which lacks petals (Di Stilio et al., 2005). The genomic locus for the *Thalictrum* ortholog of *AP3-3* is missing in *T. petaloideum* (Zhang et al., 2013), yet it remains unclear whether this loss is common to all *Thalictrum* species. The ortholog of *PI*, the other B class gene, is single-copy in diploid *T. thalictroides*, while two highly similar homeologs can be found in tetraploid *T. dioicum* (Di Stilio et al., 2005). Gene duplications affecting the *AP3* lineage of *Thalictrum* mentioned above, in combination with subtle differences in gene expression shown here, imply that some level of redundancy and of neo or sub-functionalization of paralogs may be in place. If this were the case, all three copies of *AP3* would need to be silenced in order to achieve a full B class gene knockout. For a more streamlined approach, we selected the single-copy orthologs of *PI* from *T. thalictroides* for our targeted gene silencing experiments because it was the best candidate for obtaining full B class loss-of-function phenotypes.

In the present study, we set out to recapitulate evolutionary transitions in sexual system in the ranunculid genus *Thalictrum* by tinkering with the levels of B class gene expression in dioecious and hermaphroditic species. A combination of gene expression analyses and VIGS allowed us to test the potential functional role of the orthologs of *PI* in sexual system changes. Finally, we contribute to mounting evidence of conservation of B class gene function between core and non-core eudicots, including the less reported expression of *AP3* in sepals (Weigel and Meyerowitz, 1993; Krizek and Meyerowitz, 1996).

## Results

### Characterization of thalictrum B class transcript expression

**Figure 1:**
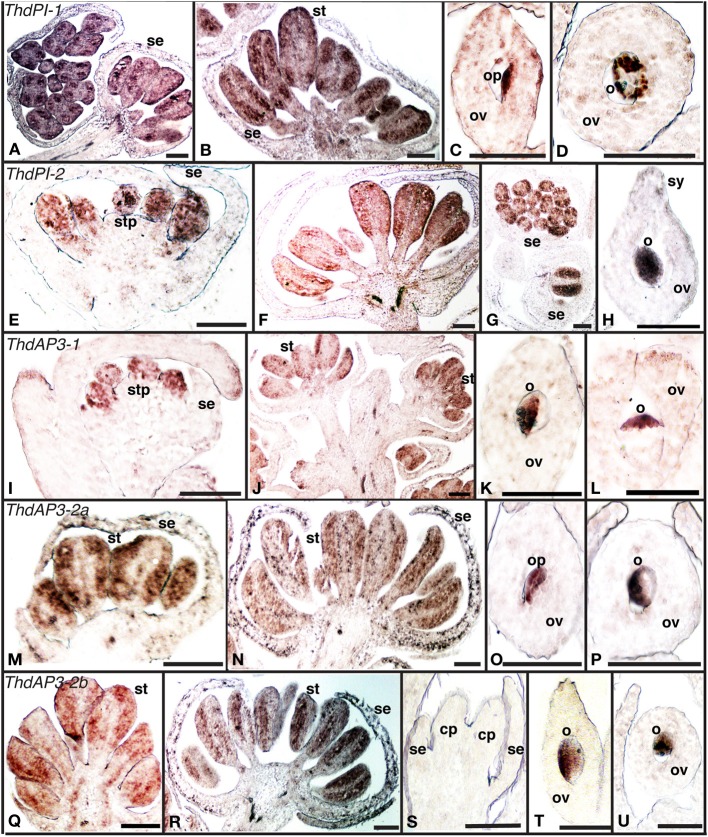
**Expression of B class gene orthologs in the dioecious species *Thalictrum dioicum* by *in situ* hybridization. (A–D)** *ThdPI-1*; **(E–H)** *ThdPI-2;* **(I–L)** *ThdAP3*-1, **(M–P)** *ThdAP3-2a*, **(Q–U)** *ThdAP3-2b*. Male flower buds: **(A**, c.s. left**)**, **(E**,**I**,**M**,**Q)**. Male open flowers: **(B**, **F**,**J**,**N**,**R**,**G)** (c.s.). Female flower carpels: **(C)**, **(D**, c.s.**)**, **(H**,**K)**, **(L**, c.s.**)**, **(O)**, **(P**, c.s.**)**, **(T)**, **(U**, c.s.**)**. Female bud, with carpel primordia: **(S)**. c.s., cross section; cp, carpel primordium; o, ovule; op, ovule primordium; ov, ovary; se, sepal; st, stamen; stp, stamen primordium; sy, style. Scale bar = 1 mm.

B class genes orthologous to *PI* and *AP3* had been previously cloned and their expression analyzed by RT-PCR in the hermaphroditic species *T. thalictroides* (*Tht*) and in the dioecious species *T. dioicum* (*Thd*). For a more detailed characterization of expression patterns in connection with sex determination, we carried out *in situ* hybridizations for the five B-class genes (*ThdAP3-1, ThdAP3-2a, ThdAP3-2b, ThdPI-1*, and *ThdPI-2*) in flowers of male and female plants of *T. dioicum* (Figure 1). In spite of moderate levels of background, signal was mostly strong and distinct (see sense controls, Figure S1A).

All B class genes were expressed in male flowers from the earliest observed stages, within stamen primordia and young stamens (Figures 1A,B,E–G,I,J,M,N,Q,R). As expected, B class genes were not expressed in female buds (Figure 1S, one shown). However, signal was detected later in female development, within the ovules of mature carpels (Figures 1C,D,H,K,L,O,P,T,U). This is consistent with ovule B class expression in other ranunculids (Kramer and Irish, 1999). In one case, expression appeared evenly throughout the ovule (*ThdPI-2*, Figure 1H), while for all other loci, ovule expression was clearly asymmetrical (Figures 1D,K,L,P,T,U). In two cases (*ThdPI-1* and *ThdAP3-2a*), gene expression could be seen in the placenta, demarcating the ovule primordium (Figures 1C,O). For those genes where very young bud sections were available (*ThdPI-2* and *ThdAP3-1*), expression in sepals was not detected during early development for any of the B class genes (Figures 1E,I). Both *ThdPI* loci had the same expression pattern (compare Figures 1A–D to E–H); their high level of sequence similarity (96% at the nucleotide level) makes them likely homeologs (Di Stilio et al., 2005) and restricted our ability to design locus-specific probes (see methods). *ThdAP3-2a and ThdAP3-2b* had identical expression patterns also (compare Figures 1M–P to Q–U); which differed from *ThdAP3-1* and *ThdP1-1/2* in that they were detected also in sepals (in the epidermis and in the inner cell layers of the sepal base) of older male buds (Figures 1N,R), but not in females (Figure S1B). In brief, B class gene expression in *T. dioicum* was overall consistent with expectations from the canonical ABC model, including late expression of two of the AP3 orthologs in sepals of male flowers. In *Arabidopsis thaliana*, *AP3* is expressed in the base of sepals of flowers at stage 6–7, with no apparent functional consequences (Weigel and Meyerowitz, 1993; Krizek and Meyerowitz, 1996). In *Thalictrum dioicum*, sepal expression seems to occur later in flower development, extending to the whole epidermis. It remains to be tested whether *ThtAP3-2a/b* expression in petaloid sepals of *T. thalictroides* (Di Stilio et al., 2005), could potentially be involved in elaboration of sepals late in development in that species.

### Experimental phenocopy of evolutionary transitions

We conducted targeted gene silencing experiments of *ThPI* by VIGS in *T. dioicum* and *T. thalictroides*, with the dual goal of (1) recapitulating putative transitions in sexual system by homeosis within *Thalictrum* and (2) testing the degree of conservation of B class gene function in a non-core eudicot.

#### VIGS of the B class gene ThdPI-1/2 in Thalictrum dioicum

**Figure 2:**
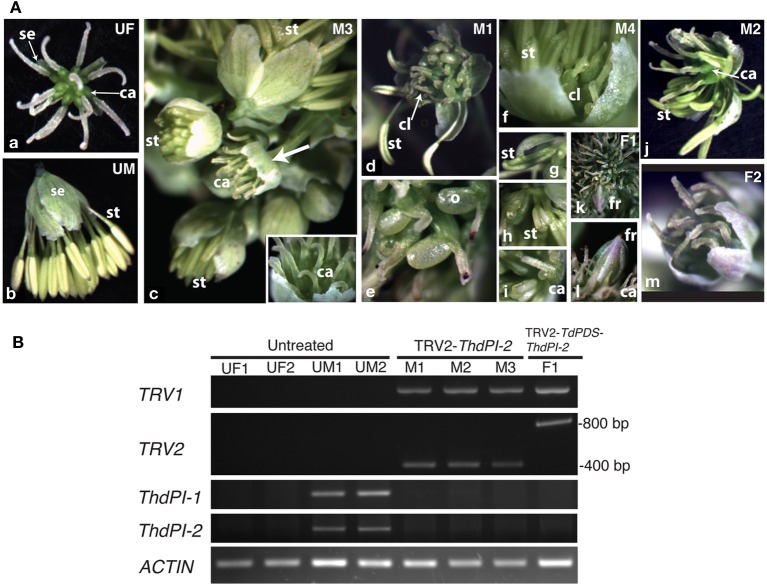
**Targeted gene silencing by VIGS of the *PI* orthologs *ThdPI-1* and *ThdPI-2* in the dioecious species *Thalictrum dioicum* causes homeotic transformation of stamen primordia into carpels, turning male flowers into female flowers. (A)** Flower phenotypes resulting from VIGS. **(a)** untreated female flower (UF); **(b)** untreated male flower (UM); **(c)** male inflorescence of TRV2-*ThdPI-2* treated plant with homeotic female flower (M3, arrow), detailed in inset; **(d)** partially homeotic male flower (M1) with few remaining normal stamens and homeotic carpel-like organs, **(e)** detail of homeotic open carpel with exposed ovule; **(f)** weakly-silenced male flower (M4) with a few carpel-like organs visible against the sepals, **(g,h)** detail of carpeloid stamens in increasing degree, **(i)** open carpeloid organ; **(j)** hermaphroditic flower (M2) with carpels in the center and stamens around; **(k)** female flower from TRV2-*TdPDS*-*ThdPI-2* treated plant (F1) with photobleached fruit, **(l)** detail of fruit and surrounding wildtype carpels; **(m)** representative treated female flower. ca, carpel; cl, carpel-like organ; fr, fruit; o, ovule; se, sepal; st, stamen. **(B)** Molecular validation of VIGS phenotypes in part A by reverse transcriptase (RT) PCR. Detection of TRV1, TRV2, and *ThdPI-1/2* transcripts are shown for four untreated plant samples (females UF1 and UF2 and males UM1 and UM2), three TRV2-*ThdPI-2* treated male flowers from one plant (M1–M3) and one TRV2-*ThdPI-2* treated female flower (F1). Approximate sizing of TRV transcripts for the single (TRV2-*ThdPI-2*) and the double (TRV2-*TdPDS*-*ThdPI-2*) constructs indicated on the right. ACTIN used as loading control.

In *T. dioicum*, untreated female flowers consist of variable numbers of small green sepals (4–5) and spirally arranged free carpels (5–17) with one apical ovule each (Figure 2Aa); male flowers consist of slightly bigger sepals in similar numbers as female flowers and numerous spirally arranged stamens [20–42, Figure 2Ab; organ count data from Di Stilio et al. (2005)]. Vacuum infiltration of plants followed by injection with TRV2-*ThdPI-2* or TRV2-*TdPDS-ThdPI-2*, resulted in the infection of two plants (one for each construct) showing male to female homeotic phenotypes and one female plant showing photobleaching but no homeotic phenotype (Table 1, Figure 2). In the strongest line, a wild-type-looking female flower developed within a male inflorescence (flower M3, Figure 2Ac, arrow and inset detail). In a partially silenced nearby flower most stamen primordia developed into carpel-like organs, while a few of the outer stamens developed normally (flower M1, Figures 2Ad,e). Within this partially silenced flower, homeotic carpels were also abnormal, with the single ovule extruded and shorter styles (Figure 2Ae). Open carpels with exposed ovules are reminiscent of those described for the *Arabidopsis thaliana* B class mutant (Bowman et al., 1989). Milder phenotypes consisted of male flowers with only a few carpel-like organs toward the periphery [flower M4, Figure 2Af, detail in (i)], and stamens with carpeloid features, such as anthers with stigmatic ends (Figures 2Ag,h). One of the flowers (M2, Figure 2Aj) developed as a hermaphrodite, with carpels in the center, surrounded by stamens. Flowers from untreated and TRV2 empty plants did not show any of the homeotic phenotypes described above (Figures 2Aa,b and not shown). Treated females did not differ from untreated female plants (Figure 2Am). A flower from an extensively photobleached female plant developed an apparently normal fruit with a photobleached fruit wall (young fruits are normally green, Figures 2Ak,l). This result suggests that, at least in this one case, ovule development does not depend on *ThdPI-1/2* expression.

**Table 1:** **Number of plants (flowers within a plant) treated, surviving, showing homeotic phenotypes, and photobleached only, after virus induced gene silencing of orthologs of the B class gene *PISTILATA* (*PI*) in *Thalictrum dioicum* (*Thd*) and *T. thalictroides* (*Tht*)**.

**Construct**	**Species**	**Treated plants**	**Surviving plants**	Homeotic plants (flowers)^*^ sex	**Photobleached plants (flowers) (no homeosis)**
TRV2-*ThdPI-2*	*T. dioicum*	41	25	1 (4) male	
TRV2-*TdPDS-ThdPI-2*	*T. dioicum*	37	34	1 (1) male	1 (1) female
TRV2 empty	*T. dioicum*	10	5	0	
Untreated	*T. dioicum*	10	7	0	
TRV2-*ThtPI*	*T. thalictroides*	29	24	4 (8)	
TRV2-*TdPDS-ThtPI*	*T. thalictroides*	65	39	2 (4)	
TRV2 empty	*T. thalictroides*	10	6	0	
Untreated	*T. thalictroides*	14	12	0	

*TRV, tobacco rattle virus; PDS, phytoene desaturase (used as photobleaching reporter gene)*.

* Validated by detection of TRV1/TRV2 transcripts.

To determine whether the observed homeotic phenotypes resulted from downregulation of *ThdPI* transcripts, we carried out RT-PCR on flowers (Figure 2B). Tissues for molecular validation were available from three male flowers showing strong to intermediate phenotypes (M1, Figure 2Ad; M2, Figure 2Aj and M3, Figure 2Ac) and one photobleached female flower (F1, Figure 2Af); all were positive for TRV1/2 transcripts (Figure 2B). The TRV2-*ThdPI-2* treated male flowers had no detectable *ThdPI-1* or *ThdPI-2* expression, while untreated male flowers expressed detectable levels of both genes (Figure 2B). In spite of a few stamens present in two treated male flowers (M1 and M2), *ThdPI-1/2* were not detected, possibly due to the low cycle number (25). Untreated female flowers had no detectable levels of *ThdPI-1/2* expression (Figure 2B) (only ovules in mature flowers express these genes, Figures 1Ac,d,h).

In summary, targeted gene silencing of both homeologous *PI* orthologs, *ThdPI-1*, and *ThdPI-2*, was confirmed in three plants of *T. dioicum*, resulting in homeotic conversions of stamen to carpel development in male plants. The resulting flowers in silenced male plants were undistinguishable from wildtype female flowers. VIGS-treated female plants had no visible phenotype.

#### VIGS of the B class gene ThtPI in Thalictrum thalictroides

**Figure 3:**
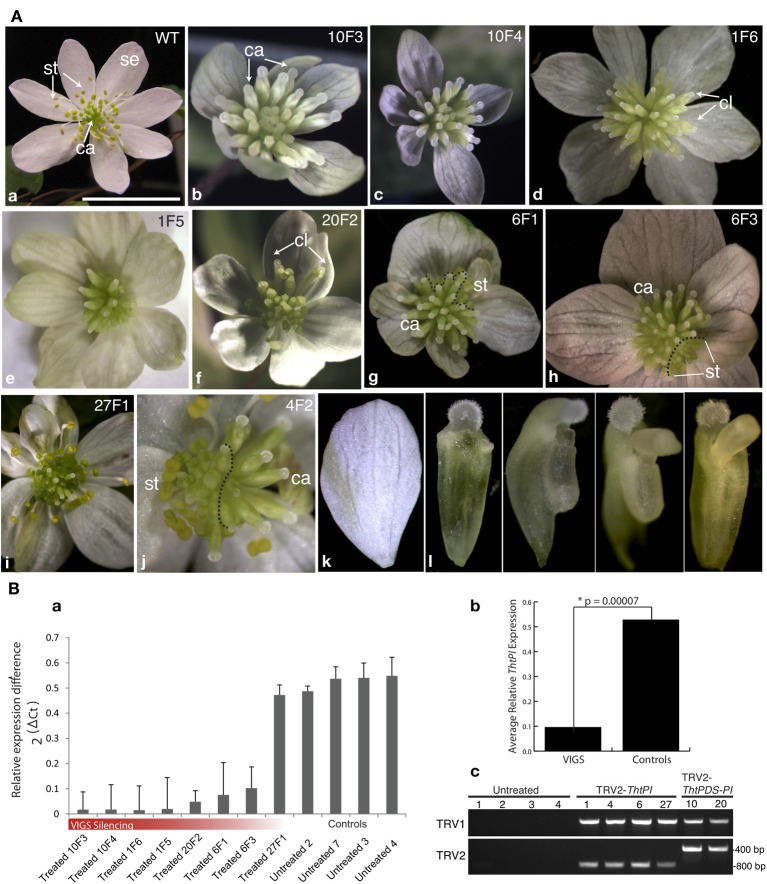
**Targeted gene silencing of the *PISTILLATA* ortholog *ThtPI* by virus induced gene-silencing (VIGS) results in homeotic transformation of stamen primordia into carpels, resulting in female flowers in the hermaphroditic species *Thalictrum thalictroides*. (A)** Flower phenotypes resulting from VIGS and untreated controls. Original sample identifier indicated on the top right (plant number, followed by flower number). **(a)** wild-type flower of *T. thalictroides*; **(b–j)** flowers from plants treated with TRV2-*ThtPI* **(d,e,g–j)** or TRV2-*TdPDS*-*ThtPI* **(b,c,f)**; **(k)** detail of chimeric organs with mixed carpel and stamen characteristics. **(g,h,j)** dashed lines delineate areas undergoing gene silencing (carpels in the periphery) from normal areas (stamens in the periphery) in chimeric flowers. **(b,c)** examples of homeotic phenotypes resulting from strong silencing of *ThtPI*, note lack of stamens and supernumerary carpels; **(d)** outer carpel-like organs (cl) with extruded ovules, **(e)** stamens reduced to sterile scale-like organs and green-tinted sepals, **(f)** another example of outer carpel-like organs (cl) with extruded ovules, **(g)** leafy sepals with green sectors on sepals, abnormal outer carpels and a few stamens present, **(h)** chimeric flower with stamens present in one sector, **(i)** mild phenotype consisting of green stripes on sepals only, **(j)** half-silenced flower, **(k)** detail of sepal with green stripe, **(l)** details of chimeric organs (from left to right): progressively more stamen-like carpels, with anther-like flaps and extruded ovule. Se, sepal; st, stamen; ca, carpel. Scale bar 1 cm. **(B)** Molecular validation of VIGS phenotypes by qPCR, arranged as in part **(A)**. **(a)** *ThtPI* expression in eight treated (TRV2-*ThtPI* or TRV2-*TdPDS*-*ThtPI*) and four untreated controls relative to averaged *TtACTIN* and *TtEEF1* standards. **(b)** Average *ThtPI* expression difference in VIGs treated plants vs. controls (relative to *TtEEF1* and *TtACTIN*), ^*^ two-tailed Student's *t*-test with unequal variance. **(c)** detection of TRV1 and TRV2 viral transcripts in six treated plants, viral transcripts could not be detected in untreated controls, two plants (last two lanes) have longer inserts in TRV2, as expected from having the double constructs TRV2-*TdPDS*-*ThtPI*.

Wild-type flowers of the hermaphroditic species *T. thalictroides* consist of 5–12 white petaloid sepals, 45–76 stamens and 3–11 free carpels (Galimba et al., 2012). Vacuum infiltration of tubers with a TRV2-*ThtPI* construct resulted in a gradient of homeotic flower phenotypes, culminating in female flowers with no remnants of stamens (Figure 3, Figure S2). These female flowers had supernumerary carpels in lieu of stamens when compared to untreated (or TRV2-empty) controls (compare Figures 3Aa to b,c). On first inspection, sepals from treated plants appeared mostly undistinguishable from untreated controls, both in number and quality. Some of the sepals within a flower were smaller than others (Figures 3g–i), but because this phenotype had been previously detected in empty TRV2 controls (Di Stilio et al., 2010), it was unclear to what extent this was a consequence of *ThtPI* silencing, as opposed to a viral background effect. On closer inspection, however, most sepals exhibited some level of “leafy” appearance, ranging from an overall green tint to distinct green sectors (Figures 3Ab,d,e,g,i,k). Some of the flowers were chimeric, exhibiting silenced sectors with carpels only, and wildtype sectors with stamens and carpels (Figures 3Ag,h,j). In one case, stamens were reduced to tiny scale-like sterile organs (Figure 3Ae). A range of stamen/carpel chimeric organs was found in intermediate to weak phenotypes (Figures 3Ad,f); Figure S2), including carpel-like organs without an ovule, with anther-like flaps, and with an extruded ovule (Figure 3Al).

TRV1 and TRV2 transcripts were detected by RT-PCR in the resulting six transgenic lines showing phenotype, while they were absent in untreated controls (Figure 3Bc). To determine whether the observed phenotypes resulted from downregulation of *ThtPI*, we carried out quantitative real-time PCR (qPCR) of eight individual flowers representing five independent VIGS events and compared them to flowers from four untreated control plants. *ThtPI* expression was downregulated over five-fold in TRV2-*ThtPI* treated plants compared to untreated controls (Figure 3Ba). The expression difference between VIGS and control plants was highly significant in a two-tailed Student's *t*-test of 2^ΔCt^ values (VIGS 0.1 ± 0.05; Untreated 0.53 ± 0.01, *p*-value = 7 × 10^−5^, Figure 3Bb). In summary, downregulation of *ThtPI* resulted in homeotic conversion of stamens to carpels and in sepals with green sectors; the presence of virus in treated plants and the downregulation or complete absence of *ThtPI* transcript suggest that this was the cause for the observed homeotic phenotypes.

## Discussion

The sexual conversions resulting from targeted silencing of the *Thalictrum* orthologs of the B class gene *PI* suggest that (1) transitions in sexual system (hermaphrodite or male to female) are possible in one step and (2) homeosis is a viable model for the evolution of dioecy in *Thalictrum.* Moreover, our expression and functional results in two ranunculid species add to growing evidence of deep functional conservation of *PI* and *AP3* orthologs in stamen identity beyond core eudicots, while divergent expression patterns of *AP3* orthologs are consistent with repeated sub-functionalization events in other ranunculids (Drea et al., 2007; Kramer et al., 2007; Sharma et al., 2011; reviewed in Di Stilio, 2011).

Whether B class genes are responsible for ectopic petaloidy seems to be lineage-specific. In our study, sepals retained overall their petaloid features in the strong phenotypes derived from VIGS in *T. thalictroides* (Figures 3Ac,f,h). This would suggest that, in spite of being expressed in sepals (Di Stilio et al., 2005), *ThtPI* is not directly or solely implicated in ectopic petaloidy in this species, as has been suggested in monocots with petaloid tepals (Kanno et al., 2003; Nakamura et al., 2005). Yet, upon closer inspection, most sepals in TRV2-*ThtPI* treated plants had green sectors (Figures 3Ab,d,e,g,i,k), and some were considerably smaller (Figures 3Ah–j), but the latter could be a viral effect, see results). Green sepals in silenced flowers suggest a role of *ThtPI* in suppressing chlorophyll, contributing to ectopic petaloidy. In the closely related *Aquilegia vulgaris*, which has large and brightly colored petaloid sepals, silencing of the *PI* ortholog, *AqvPI*, also results in smaller and greener sepals, although this effect may occur via the associated downregulation of the *AP3-2* genes (Kramer et al., 2007). In fact, the petal identity network in *Arabidopsis thaliana* involves the suppression of green pigment by both *AP3* and *PI* from otherwise leaf-like organs (Mara et al., 2010). Therefore, the effect of *ThtPI* on petaloidy of *T. thalictroides* flowers could be indirect, via downregulation of the presumably interacting partners ThtAP3-2a and ThtAP3-2b (we did not assess the expression of *AP3* orthologs in our VIGS plants). Alternatively, petaloidy in *Thalictrum* may be independent of B class gene function, as has been found in other systems (Landis et al., 2011).

The role of B (and C) class genes in sex determination has been functionally tested in one other Type II floral system, spinach, which is dioecious by suppression of organ primordia, rather than by homeosis (Sather et al., 2010). Similar to our study, male plants undergoing gene silencing of B class genes were capable of producing wild-type looking female flowers, and the model proposed for the evolution of dioecy in spinach also involved regulation of B class genes.

Our recapitulation of the evolution of female flowers from hermaphrodites, a step in the evolution of dioecy, was conducted using the insect-pollinated species *T. thalictroides*. This scenario assumes dioecy evolving before wind pollination, a sequence that has been reported across angiosperms (Friedman and Barrett, 2008). Floral traits that are typical of the wind pollination syndrome and present in species like *T. dioicum*, such as pendulous male flowers, reduced sepals and extended stigmatic surfaces (Figures 2Aa,b), would have had to evolve after the B gene mutations. Except for a tendency to greener and occasionally smaller sepals, silencing of *ThtPI* did not result in wind pollination floral traits, so these would have had to evolve subsequently. It is interesting to note that showy flowers of *Aquilegia vulgaris* experiencing strong silencing of *AqvPI* (Kramer et al., 2007) are remarkably similar to the green and inconspicuous flowers of *T. dioicum*, demonstrating that loss of insect pollination syndrome traits can happen quickly and by the action of a limited number of genes (in this case, just one gene). The experiments on *T. dioicum*, on the other hand, demonstrate that reversals in sexual system could have occurred starting from a wind-pollinated species. Given that wind pollination and dioecy tightly coevolved within *Thalictrum*, it is difficult to infer which originated first; although there is some indication that wind pollination evolved early within the genus (Soza et al., 2012).

**Figure 4:**
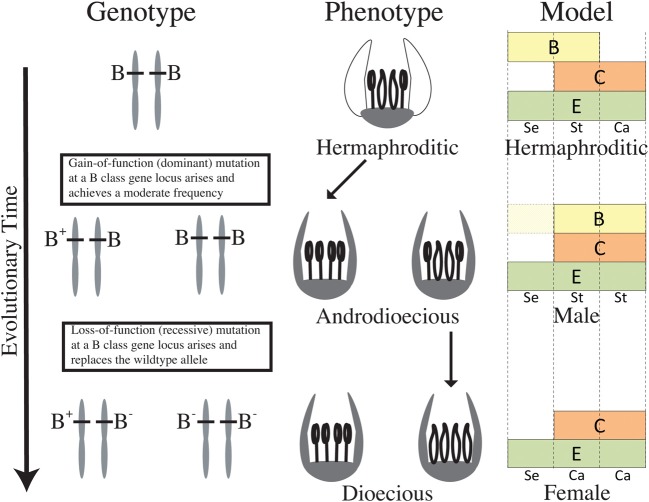
**Hypothetical model for the evolution of dioecy from an insect pollinated hermaphrodite via androdioecy in *Thalictrum*, based on functional data presented here and prior phylogenetic reconstructions of sexual system (Soza et al., 2012).** Modified “ABC” model of a hypothetical hermaphroditic ancestor (based on functional and expression data for *T. thalictroides*, this study and Di Stilio et al., 2005) and its dioecious descendants (based on *T. dioicum* expression and functional data, this study). Petals have been lost in this genus, therefore only sepals (Se), stamens (St), and carpels (Ca) are represented. “A” class is not included, given mounting evidence that it does not hold beyond Brassicaceae (Litt, 2007; Causier et al., 2010). A gain-of-function mutation in a B class gene of a hermaphroditic ancestor extends B function to the carpel zone, causing homeotic conversion of carpels to stamens and resulting in individuals with male flowers. A subsequent loss-of-function mutation at the same B class locus results in homeotic conversion of stamens to carpels resulting in individuals with female flowers. B class genes are also expressed in petaloid sepals of the hermaphroditic ancestor where their products have a potential function in suppressing chlorophyll. B class genes are only expressed late in development of male sepals in the dioecious descendant, with no apparent functional consequences (represented by lighter yellow area). E class function is depicted throughout the floral meristem, as describe in most of flowering plants surveyed (Malcomber and Kellogg, 2005).

In light of recent reconstructions of sexual system evolution in *Thalictrum* (Soza et al., 2012, 2013), dioecy evolved twice from hermaphroditism. The clade containing *T. dioicum* is closely related to an andromonoecious clade, while the other dioecious clade has at least two structurally androdioecious species nested within it (Soza et al., 2012). The absence of gynodioecy in extant taxa and the rare presence of gynomonoecy in two isolated species suggest that dioecy most likely evolved via loss of female function in *Thalictrum*, followed by loss of male function. Based on functional data presented here, we hypothesize that a B class gene may have been a likely target of the mutations leading to dioecy in *Thalictrum*. If sex determination is homeotic-like, the two required mutations for the evolution of dioecy, one for female and one for male sterility (Charlesworth and Charlesworth, 1978), can occur at a single locus. Our model for the putative role of B class genes in the evolution of dioecy in *Thalictrum* starts with a gain of function mutation in a hermaphroditic ancestor, resulting in an androdioecious population (Figure 4, top). As mentioned above, this rare sexual system exists, at least structurally, in two extant species (*T. pubescens* and *T. macrostylum*), embedded within one of the dioecious clades (Soza et al., 2012). A second step would have involved a loss of function mutation at the same locus, causing female flowers to replace hermaphrodites and resulting in a dioecious population (Figure 4, bottom). The model may be similarly applied to the andromonoecy pathway, with mutations at the B locus differentially affecting its regulation in floral meristems within a plant. The proposed model would gain additional support if (1) sex-linked alleles were found at one of the B loci, and (2) a stable transgenic system allowed the overexpression of *ThtPI* to recapitulate the gain of function step to maleness. Future directions should also include loss and gain of B function experiments in a wind-pollinated hermaphrodite.

## Methods

### Plant material

*Thalictrum dioicum* plants used in this study were propagated at the University of Washington (UW) greenhouses from wild-collected plants [V. Di Stilio 101 (A)]. *Thalictrum thalictroides* plants were bought from local nurseries and subsequently propagated in the greenhouse [V. Di Stilio 124 (WTU)].

### Expression of B class genes in thalictrum dioicum by *In situ* hybridization

*In situ* hybridizations were performed as described in Kramer (2005) with minor modifications. Gene-specific probes excluded regions of high similarity between the three *Thalictrum AP3* and the two *PI* loci. Probes were approximately 400 bp. long, including half their length of coding region and half of 3′ untranslated region (3′UTR). In the case of the two *Thd PI* homeologous loci, probes were 72% similar, and cross-hybridization is a possibility. All sequences of B class loci for both species had been previously reported (Kramer et al., 2003; Di Stilio et al., 2005).

### Construct preparation for VIGS

A 400 bp. region of *ThdPI-2* equivalent to the *in situ* probe and containing 200 bp. of the end of the coding region and 200 bp. of the beginning of 3′UTR was amplified from existing plasmid (see Table S1 for primers) and cloned into the TRV2 multiple cloning site (Liu et al., 2002). This region is highly homologous in the two genes, *ThdPI-1* and *ThdPI-2*, and was designed to silence both loci at once. For the single copy *ThtPI*, the silencing fragment was selected more upstream, including the last 400 bp. of coding region. The *T. dioicum* and *T thalictroides* PDS loci (Genbank FJ457899, HM488111) are 99% identical at the nucleotide level over the silencing fragment. Therefore, we used the available TRV2-*TdPDS* as a starting point for both double constructs. The respective silencing fragments of *ThdPI-2* or *ThtPI-2* were cloned into pTRV2-*TdPDS* (Di Stilio et al., 2010).

### Virus induced gene silencing of ThtPI in *Thalictrum thalictroides*

Tubers of dormant *T. thalictroides* plants were treated by agroinfiltration under vacuum as described previously (Di Stilio et al., 2010). Two constructs were used: the double construct TRV2-*TdPD*S-*ThtPI* including the “marker” gene *Phytoene desaturase* that causes visible photobleaching of photosynthetic tissues, and the TRV2-*ThtPI* single construct containing a fragment of the target gene only. Empty TRV2 was used as a control for virus-related phenotypes. Plants were co-infected with one of the TRV2 constructs and TRV1.

### Virus induced gene silencing of *ThdPI-1* and *ThdPI-2* in *T. dioicum*

Similar to *T. thalictroides*, we used two experimental constructs in *T. dioicum*: TRV2-*TdPDS*-*ThdPI-2* and TRV2-*ThdPI-2*, a TRV2-empty control and TRV1. Because this species proved recalcitrant to VIGS of floral tissues, we tried two approaches to deliver the constructs: (1) agroinfiltration of seedlings under vacuum as described previously (Di Stilio et al., 2010), (except that plants were older, 2.5 months old rather than seedlings), followed by injection of 6 weeks later (closer to flowering), and (2) agroinfiltration of bare root plants (rhizomes) under vacuum, as previously described for other species of *Thalictrum* (Di Stilio et al., 2010), with 5 min vacuum time. Only the first approach yielded results (Table 1).

### Imaging of VIGS phenotypes

Flowers of both species were photographed using a dissecting microscope (Nikon SMZ800, Nikon Instruments Inc. Melville, NY) equipped with a QImaging MicroPublisher 3.3 RTV digital camera (Surrey, BC, Canada). Images were processed in Adobe^®^ Photoshop^®^ CS5 v. 12.0.2 and figures were assembled in Adobe^®^ Illustrator^®^ v. 15.0.2. Flowers were subsequently flash-frozen in liquid Nitrogen for molecular validation.

### Molecular validation of VIGS experiments by RT-PCR and qPCR

Total RNA was prepared from 50 to 100 mg of frozen floral tissue using Trizol (Invitrogen, Carlsbad, CA), following manufacturer's instructions. One microgram of the resulting total RNA was treated with amplification grade DNase I (Invitrogen) to eliminate potential genomic contamination, reverse-transcribed to cDNA using Superscript III first-strand synthesis kit (Invitrogen) with Oligo(dT)_20_ or primers specific to TRV1 (OYL 198) or TRV2 (pYL156R, Hileman et al., 2005).

To test for presence of the TRV1 and TRV2 viral RNA in treated plants, reverse-transcriptase (RT)-PCR was carried out on 1 μl of cDNA using TRV1-specific primers OYL195/198 and TRV2-specific primers pYL156F/R (Hileman et al., 2005), for 30 cycles at 51°C.

Quantification of *ThdPI-1/2* expression was performed using RT-PCR, as previously described (Di Stilio et al., 2010). *TdACTIN* was amplified for 25 cycles at 58°C with primers TthACTIN for2/rev2 (Di Stilio et al., 2010) and *ThdPI-1/2* for 25 cycles at 50°C with locus-specific primers ThdPI-1F/R and ThdPI-2F/R (Di Stilio et al., 2005).

Quantification of *ThtPI* expression was performed using qPCR, as previously described (Galimba et al., 2012). Briefly, each 30 μl reaction contained 15 μl of SYBR Green PCR Master Mix (Bio-Rad), 0.9 μl (10 μM) of locus-specific primers (Table S1), 1 μl of template cDNA and 12.2 μl of water. Samples were amplified for 40 cycles in triplicate, including a no-template control, under the following conditions: 94°C for 10 min, 45 cycles of 94°C for 30 s, 54°C for 30 s, and 72°C for 30 s on the MJ Research Chromo4 PCR system (Waltham, MA, USA) at the Comparative Genomics Center (UW). Reactions were normalized to the *Thalictrum* orthologs of two housekeeping genes, *ACTIN* and *EEF1* (*EUKARYOTIC ELONGATION FACTOR 1*), using the ΔCT relative quantification method (Livak and Schmittgen, 2001). Average values and standard errors were graphed, and compared statistically by two-tailed Student's *t*-test with unequal variance.

### Conflict of interest statement

The authors declare that the research was conducted in the absence of any commercial or financial relationships that could be construed as a potential conflict of interest.

## Supplementary material

Table S1 **Newly reported primers used to build TRV constructs for VIGS and for molecular validation**.

Figure S1 **(A)** Sense controls for *in situ* hybridization experiments on B class genes in *Thalictrum dioicu* male buds. The probe is indicated in each panel. **(B)** *In situ* hybridization of *ThdAP3-2a* and *ThdAP3-2b* on female buds of *T. dioicum*, and sense control.

Figure S2 **Additional chimeric flowers resulting from VIGS of *ThtPI* in *Thalictrum thalictroides*, belonging to plants used in the molecular validation experiments.** Sample identifier (plant number, followed by flower number) indicated on the top right (refer to Figure 3). **(A)** outer stamens replaced by chimeric carpel-like (cl) organs; **(B)** detail of floral center in **(A)**; **(C)** two stamens (arrowheads) and 2 filamentous organs (asterisks) visible outside of carpel-like organs; **(D)** mild phenotype, with five carpel-like organs (cl) in place of stamens in the flower periphery.
