# Supplementary material for: Functional recapitulation of transitions in sexual systems by homeosis during the evolution of dioecy in Thalictrum
Source: Front Plant Sci. 2013 Nov 27;4:487. doi: 10.3389/fpls.2013.00487 (PMC3842162; doi:10.3389/fpls.2013.00487)
Supplement: Table S1 — Newly reported primers used to build TRV constructs for VIGS and for molecular validation. [file DataSheet1.PDF]

Species	Locus	Primer name	Primer sequence	Purpose
Thalictrum thalictroides	*ThtPI*	ThtPI_fwd_BamHI	5’AGTGGATCCATCAGCCCTTC TCCTGGTCC 3’	VIGS construct
		ThtPI_rev_XhoI	5’AACTCGAGATCATTCCCGCC GATTTC 3’	
		ThtforqPCR	5’AAGTATGCTAGAGGAAGAG AACAAGC 3’	qPCR
		ThtPIrevqPCR	5’CTCATGGTAGGTGATACTTTC GTTCTA 3’	
Thalictrum dioicum	*ThdPI-2*	ThdPI-2-F-EcoRI	5’GCGAATTCCATGATGAGCAA AAATACAGC 3’	VIGS construct
		ThdPI-2-R-BamHI	5’AGGGATCCAGAGGGCGAAT ACCAAAC 3’	

Suppl. Table 1 (Table 2): Newly reported primers used to build TRV constructs for VIGS and for molecular validation.
