# Supplementary figures and images for: Functional recapitulation of transitions in sexual systems by homeosis during the evolution of dioecy in Thalictrum
Source: Front Plant Sci. 2013 Nov 27;4:487. doi: 10.3389/fpls.2013.00487 (PMC3842162; doi:10.3389/fpls.2013.00487)

A

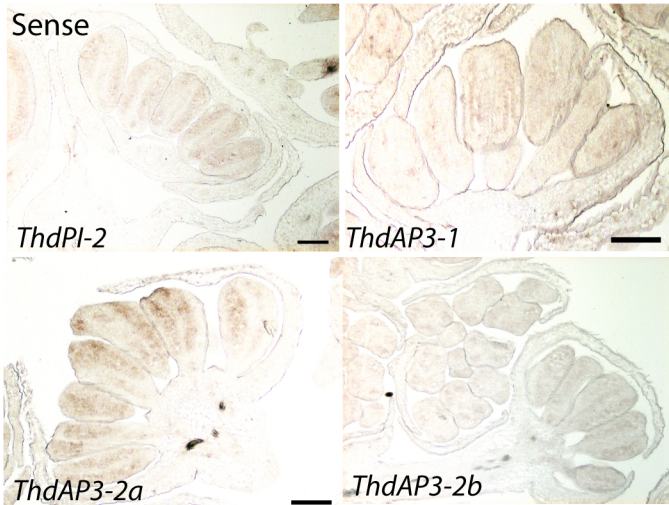

B

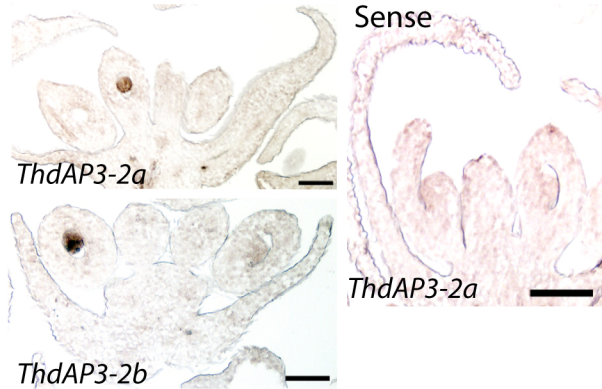

Supplement: Figure S1 — (A) Sense controls for in situ hybridization experiments on B class genes in Thalictrum dioicu male buds. The probe is indicated in each panel. (B) In situ hybridization of ThdAP3-2a and ThdAP3-2b on female buds of T. dioicum, and sense control. [file Presentation1.PDF]

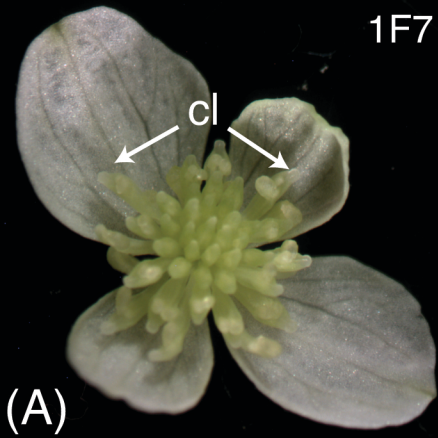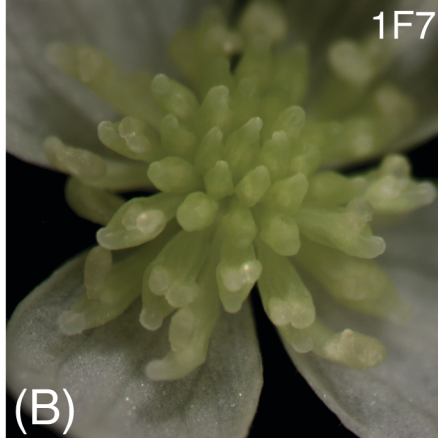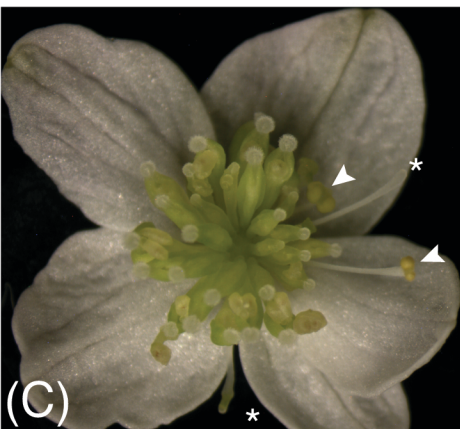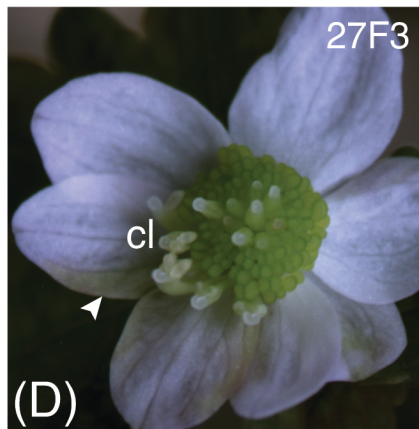

Supplement: Figure S2 — Additional chimeric flowers resulting from VIGS of ThtPI in Thalictrum thalictroides, belonging to plants used in the molecular validation experiments. Sample identifier (plant number, followed by flower number) indicated on the top right (refer to Figure 3). (A) outer stamens replaced by chimeric carpel-like (cl) organs; (B) detail of floral center in (A); (C) two stamens (arrowheads) and 2 filamentous organs (asterisks) visible outside of carpel-like organs; (D) mild phenotype, with five carpel-like organs (cl) in place of stamens in the flower periphery. [file Presentation2.PDF]
